# GEMMs as preclinical models for testing pancreatic cancer therapies

**DOI:** 10.1242/dmm.021055

**Published:** 2015-10-01

**Authors:** Aarthi Gopinathan, Jennifer P. Morton, Duncan I. Jodrell, Owen J. Sansom

**Affiliations:** 1 Cancer Research UK Cambridge Institute, University of Cambridge, Robinson Way, Cambridge, CB2 0RE, UK; 2 Cancer Research UK Beatson Institute, Glasgow, G61 1BD, UK

**Keywords:** Co-clinical trials, Preclinical mouse models, Pancreatic ductal adenocarcinoma, PDAC, Drug discovery, Drug development

## Abstract

Pancreatic ductal adenocarcinoma is the most common form of pancreatic tumour, with a very limited survival rate and currently no available disease-modifying treatments. Despite recent advances in the production of genetically engineered mouse models (GEMMs), the development of new therapies for pancreatic cancer is still hampered by a lack of reliable and predictive preclinical animal models for this disease. Preclinical models are vitally important for assessing therapies in the first stages of the drug development pipeline, prior to their transition to the clinical arena. GEMMs carry mutations in genes that are associated with specific human diseases and they can thus accurately mimic the genetic, phenotypic and physiological aspects of human pathologies. Here, we discuss different GEMMs of human pancreatic cancer, with a focus on the Lox-Stop-Lox (LSL)-*Kras^G12D^;* LSL-*Trp53^R172H^; Pdx1-cre* (KPC) model, one of the most widely used preclinical models for this disease. We describe its application in preclinical research, highlighting its advantages and disadvantages, its potential for predicting clinical outcomes in humans and the factors that can affect such outcomes, and, finally, future developments that could advance the discovery of new therapies for pancreatic cancer.

## Abstract (precis)

**Drug Discovery Collection:** This Review discusses GEMMs of human pancreatic cancer, particularly focusing on the KPC model, its use in preclinical research, advantages and disadvantages, and its potential for predicting clinical outcomes.

## Introduction

Pancreatic cancers are a group of diseases that affect both the endocrine and exocrine compartments of the pancreas. The most common of these is pancreatic ductal adenocarcinoma (PDAC), an exocrine malignancy that accounts for >90% of all cases of pancreatic cancer (Feldmann and Maitra, 2008; Warshaw and Fernandez-del Castillo, 1992). PDAC is a lethal disease, with a 5-year survival rate of <5% (Hidalgo, 2010), and is the fourth leading cause of cancer-related deaths in the United States, with 48,960 new cases and 40,560 deaths estimated in 2015 (Siegel et al., 2015; Warshaw and Fernandez-del Castillo, 1992). In the United Kingdom, it is the fifth most common cause of cancer-related mortality, with 8773 newly diagnosed cases in 2011 and 8662 deaths in 2012 (Cancer Research UK, http://www.cancerresearchuk.org/cancer-info/cancerstats/types/pancreas/; accessed August 2015).

**Fig. 1.**
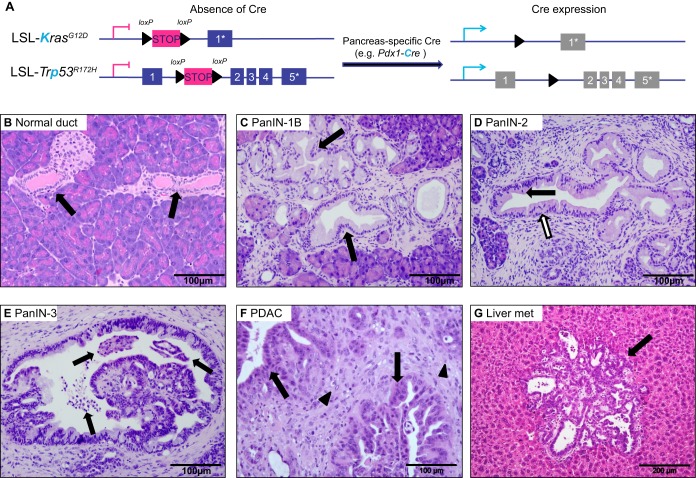
**Progression** **of pancreatic cancer in the KPC model recapitulates the human disease.** (A) In KPC mice, the conditional expression of mutant *Kras^G12D^* and *Trp53^R172H^* is controlled by a pancreas-specific *Cre* (*Pdx1-cre* in the model described here). In the absence of Cre, a transcriptional and translational STOP cassette flanked by *loxP* sites (LSL) silences the expression of mutant *Kras^G12D^* and *Trp53^R172H^*. When Cre is expressed in the pancreas, the STOP cassette is excised and the mutant alleles are expressed. The coloured boxes represent exons, and the asterisk (*) indicates the exon in which the mutation is present. The concomitant expression of mutant *Kras* and *Trp53* in the murine pancreas results in pre-invasive pancreatic intraepithelial neoplasia (PanIN), which progress to pancreatic ductal adenocarcinoma (PDAC). (B) Haematoxylin and eosin (H&E) staining of normal pancreatic ducts in wild-type mice showing that they consist of a single layer of flat, low cuboidal epithelial cells with basal nuclei (arrows). (C-G) H&E staining in KPC mice. (C) In PanIN-1B, papillary or micropapillary projections develop in the ducts (arrows), otherwise these lesions are similar to PanIN-1A (not shown here). (D) More advanced PanIN-2 is characterized by nuclear abnormalities, including loss of polarity (black and white arrows), nuclear overcrowding, enlarged nuclei and rare mitoses (white arrow). (E) PanIN-3, ductal carcinoma *in situ*, is the highest grade of neoplasm and is associated with several abnormalities, including: papillary architecture with loss of nuclear polarity; occasional aberrant mitoses; nuclear abnormalities; large prominent nucleoli; and cribriforming (small clusters of epithelial cells budding into the lumen and necrosis in the lumen) (arrows). (F) PDAC, the resulting carcinoma, exhibits a glandular phenotype with duct-like structures of varying degrees of differentiation, and can exhibit adenosquamous or sarcomatoid histology. Substantial nuclear abnormalities occur and glands appear embedded in the tumour stroma (arrowheads) with completely random organization (arrows). Tumour cells can be observed next to arteries, with perineural and vascular invasion often seen. Necrotic debris can be seen in the lumen of the gland. (G) In advanced disease, metastatic spread is common, particularly to the liver (Liver met). Metastases often exhibit a glandular histology similar to well-differentiated PDAC. Arrow shows a metastatic deposit in mouse liver.

In humans, the most frequent genetic alteration that underlies PDAC is an activating mutation of the *KRAS* oncogene (see Box 1), which occurs in >90% of tumours (Almoguera et al., 1988; Biankin et al., 2012). In addition, inactivation of the cyclin-dependent kinase inhibitor 2A (*CDKN2A*) locus, point mutations in tumour protein p53 (*TP53*) and mutations or deletions of SMAD (Sma/mothers against decapentaplegic) family member 4 (*SMAD4*) are commonly found in PDAC tumours (see Box 1) (Hruban et al., 2001b). Disease progression occurs through a series of pre-invasive pancreatic intraepithelial neoplasia (PanIN), which are graded according to their severity of dysplasia and nuclear atypia (see Box 2) (from PanIN-1, the least severe grade, to PanIN-3, which is considered ductal carcinoma *in situ* and is the last grade before invasive carcinoma). These neoplasia grades are also well replicated in animal models (Fig. 1) (Hruban et al., 2001a, 2004). Histologically, PDACs are primarily glandular, although sarcomatoid, colloid and adenosquamous (see Box 2) tumours also occur (Hruban and Adsay, 2009). These tumours are characterized by a dense desmoplastic stroma, consisting of extracellular matrix proteins – such as collagens, laminin and fibronectin – together with fibroblasts and immune cells (Adler, 2004). Early dissemination is also a common feature of PDAC, with primary tumours exhibiting perineural, vascular and lymphoid invasion (Hezel et al., 2006).

**Box 1. Commonly mutated genes in human pancreatic cancer**

***KRAS* (Kirsten rat sarcoma viral oncogene homolog)**

The *KRAS* gene encodes a protein that plays an essential role in cell signalling in normal tissue, through its activity as an ‘on/off’ switch for many signal transduction pathways, particularly those regulating cell division. Activating mutations in pancreatic tumorigenesis cause Kras to be constitutively active, which makes cells grow and divide in an uncontrolled manner.

***CDKN2A* (cyclin-dependent kinase inhibitor 2A)**

The *CDKN2A* locus is responsible for encoding two tumour suppressor proteins: p16^INK4a^ and p14^ARF^. p16^INK4a^ blocks aberrant cell growth and division. Inactivating mutations thus allow cells to grow and divide in an uncontrolled manner. p14^ARF^ protects the p53 tumour suppressor protein from being degraded (see below).

***TP53* (tumour protein p53), inactivating point mutation**

p53 is a tumour suppressor protein that stops cells from dividing too fast, or causes damaged or mutated cells that might otherwise become tumour cells to undergo apoptosis (cell suicide). It is often mutated in pancreatic tumours, meaning that mutated cells do not undergo apoptosis and that unregulated cell division occurs instead.

***SMAD4* (Sma/mothers against decapentaplegic family member 4), inactivating mutation or deletion**

The SMAD4 protein is a transcription factor (it regulates transcription of other genes) activated by signalling from the membrane-bound TGFβ (transforming growth factor beta) protein. The TGFβ pathway carries signals from the extracellular environment to the nucleus and affects how cells respond to such signals by inducing the production of new proteins. *SMAD4* deletions in pancreatic tumorigenesis can cause cells to proliferate in an uncontrolled manner.

***BRCA2* (breast cancer 2, early onset)**

The *BRCA2* gene encodes the breast cancer type 2 susceptibility protein (BRCA2). It is essential for the repair of double-stranded DNA breaks by homologous recombination, and its loss results in chromosomal instability.

**Box 2. Glossary of terms**

**Adenosquamous:** refers to a cancer type containing both gland-like cells and squamous cells (very thin, flattened cells).

**Anaplastic:** cells with abnormal morphology and loss of ordered orientation, compared to normal differentiated cells.

**Cachexia:** a wasting syndrome characterized by loss of weight, fat and muscle mass that is not reversed nutritionally.

**CD3:** a cell surface molecule that associates with the T-cell receptor to allow its activation. The presence of CD3 on the surface of all T cells at all stages of development makes it a good marker for T cells in tissue sections.

**Colloid:** glue like – refers to highly mucinous tumours (mucins are glycoproteins found in secretions).

**CRISPR/Cas:** a system used for gene editing and gene regulation. The Cas9 protein causes DNA breaks, and CRISPRs (clustered regularly interspaced short palindromic repeats) are stretches of DNA with a spacer that target a specific gene. Delivery of CRISPR guide RNAs and Cas9 into a cell allows the genome to be cut at the desired location.

**Dysplasia:** the enlargement of a tissue or organ by the proliferation of abnormal cells.

**Flp-*FRT* recombinase system:** a site-specific recombination system used to control the spatial expression of transgenes. It consists of flippase (Flp) recombinase, which targets FLP recombinase target (*FRT*) sequences that are placed at either end of a gene or region of interest.

**Fluorouracil:** an antimetabolite chemotherapeutic agent that is a pyrimidine analogue that irreversibly inhibits the nucleotide synthetic enzyme thymidylate synthase.

**Gemcitabine:** a deoxycytidine analogue used as the standard of care for pancreatic cancer. It is activated by phosphorylation, and the di- and tri-phosphate forms are responsible for its cytotoxicity. It inhibits ribonucleotide reductase, and also competes with deoxycytidine 5-triphosphate (dCTP) for incorporation into DNA during replication. It causes DNA damage and induces apoptosis.

**Haemorrhagic ascites:** the accumulation of blood-stained fluid in the peritoneum.

**Irinotecan:** a cytotoxic chemotherapeutic that inhibits topoisomerase 1, resulting in inhibition of DNA replication and transcription.

**Laparotomy:** a surgical incision in the abdominal wall to gain access into the abdominal cavity.

**Nanoparticle albumin-bound paclitaxel (nab-paclitaxel):** a paclitaxel formulation that uses albumin, the main protein of human blood plasma, to bind paclitaxel and to facilitate its transport out of the bloodstream and into the tumour. Studies show that this formulation increases the therapeutic efficacy of paclitaxel compared to its conventional formulation.

**Nuclear atypia:** abnormal cell nuclei, e.g. often by size, shape or staining pattern.

**Oxaliplatin:** a platinum-based chemotherapeutic agent. It leads to cross-linking of DNA, thereby inhibiting DNA synthesis and transcription.

**Sarcomatoid:** a histological tumour subtype characterized by spindle-shaped tumour cells.

**Transposon (or transposable element):** a short DNA sequence that can alter its position within the genome, thereby causing genetic changes.

**Xenograft:** model generated by the injection or implantation of human cancer cells or tissues into ectopic or orthotopic sites to generate tumours in immunocompromised mice.

The poor prognosis associated with PDAC can mostly be attributed to the lack of its early detection. At first diagnosis, most affected individuals present with advanced and metastatic disease, with less than 20% of patients diagnosed with resectable tumours (Heestand et al., 2015). Metastatic pancreatic cancer is associated with a median survival of less than 6 months on gemcitabine-based chemotherapy (Hidalgo, 2010) (Box 2). Gemcitabine was approved as a standard of care for treating this cancer because it provided a modest survival benefit and improvements in quality of life, compared to treatment with another chemotherapeutic, 5-fluorouracil, in a Phase 3 study (Burris et al., 1997) (Box 2). More recently, Phase 3 studies have demonstrated that the chemotherapy combinations of FOLFIRINOX (Fluorouracil/Oxaliplatin/Leucovorin/Irinotecan) and gemcitabine/nanoparticle albumin-bound (nab)-paclitaxel (see Box 2), result in improved survival over treatment with gemcitabine alone (Conroy et al., 2011; Goldstein et al., 2015; Von Hoff et al., 2013). In late 2013, the combination of gemcitabine and nab-paclitaxel was approved by the Food and Drug Administration (FDA) in the United States for the first-line treatment of metastatic PDAC. Although FOLFIRINOX and gemcitabine/nab-paclitaxel are promising recent developments in the treatment of PDAC, their benefit in terms of survival is limited to months, and therefore there is still a need to develop other novel drugs and combinations to treat this disease.

**Fig. 2.**
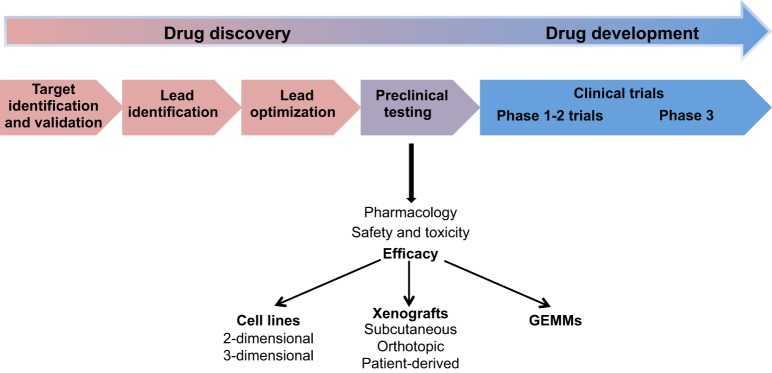
**Schema of the drug discovery and development pipeline.** The initial stages of the pipeline focus on the discovery and validation of newly identified disease-associated drug targets. Target validation is carried out using *in vitro* and *in vivo* approaches to confirm the relevance of the target in the disease being studied. This is followed by the identification of lead compounds through high-throughput or focused screens of chemical libraries or of naturally occurring molecules, or by structure-based rational drug design. The next stage is lead optimization, where the identified compound is subjected to chemical modifications to improve its pharmacological properties. Optimized lead compounds are then carried forward into preclinical testing, where pharmacology, toxicity and efficacy are assessed. Preclinical testing can occur *in vitro*, in 2- or 3-dimensional cell culture assays, or *in vivo*, in either xenografts or animal models, including genetically engineered mouse models (GEMMs). Promising therapies identified here are taken forward into the clinic. Preclinical models thus provide a bridge to the clinic, and are a requisite part of the drug development pipeline. For more detailed descriptions of the different stages of this pipeline, we refer readers to several recent reviews (Herter-Sprie et al., 2013; Hughes et al., 2011; Kamb et al., 2007; Kenakin, 2003; Pritchard et al., 2003).

Novel therapies are identified through the drug discovery and development process, which is outlined in Fig. 2. In this process, the preclinical phase acts as a bridge to the clinic, allowing promising compounds identified at earlier stages to be tested for their pharmacology, toxicity and efficacy. The successful evaluation of therapies in the preclinical setting greatly depends on the robustness and predictive ability of preclinical models, which include both *in vitro* and *in vivo* systems (Fig. 2). However, tumour complexity is not accounted for in *in vitro* systems, although co-culture models have been developed that, for instance, involve culturing cancer cells with fibroblasts, immune cells or endothelial cells (Wilding and Bodmer, 2014). *In vitro* drug testing also does not account for the effect of pharmacokinetics and drug metabolism on the activity of a compound nor for its toxicity. Historically, *in vivo* anti-cancer drug evaluation has been carried out in xenograft models (see Box 2), which can be easily and rapidly generated in immunodeficient mice by the implantation of tumour cells or of tissues into ectopic or orthotopic sites (Richmond and Su, 2008). More recently, patient-specific xenografts, which replicate features of individual patient tumours, have been developed to evaluate personalized treatment options (Rubio-Viqueira et al., 2006; Shu et al., 2008; Siolas and Hannon, 2013). Although of lower cost to generate, useful for higher throughput approaches, and complementary to genetically engineered models in comparing mouse and human tumours, xenograft models lack a functional immune system, and produce tumours of reduced complexity and cellular diversity, which could contribute to the fact that drug test results obtained in xenograft systems (as well as in *in vitro* systems) do not correlate well with efficacy testing in humans. In fact, only a small percentage of cancer patients in Phase 1 clinical trials respond to therapies as predicted (Roberts et al., 2004). The disparity between preclinical data and clinical studies can be attributed to various factors, including differences in pharmacokinetics, pharmacodynamics and metabolism, as well as a failure to accurately model the tumour microenvironment (Becher and Holland, 2006; Gopinathan and Tuveson, 2008; Sharpless and Depinho, 2006; Zhang et al., 2013). In pancreatic cancer, in particular, tumours are demonstrably stromal in nature, and the complex interactions between tumour and stromal cells might alter the efficacy of therapeutic agents. The desmoplastic stroma might also act as a barrier to the delivery of agents, such as gemcitabine (Olive et al., 2009), or as a source of survival cues that confer resistance to therapy (Vonlaufen et al., 2008; Xu et al., 2014).

Genetically engineered mouse models (GEMMs) offer an alternative to *in vitro* and xenograft models, and are currently being used to study tumour biology and responses to therapy. Mice are the preferred species for genetic manipulation because of their genetic tractability and because mice carry 99% of the same genes as humans (Mouse Genome Sequencing et al., 2002). GEMMs exist for several epithelial tumour types, including prostate, lung, breast, colon and pancreatic cancers (Frese and Tuveson, 2007). They are generally developed through the introduction of genetic mutations either in oncogenes or tumour suppressors that are associated with specific tumour types, often using conditional strategies that allow for tissue-specific regulation of these genes. GEMMs can therefore faithfully recapitulate some human cancers in terms of their genetics and phenotype. They can thus be used to study tumour biology, initiation and progression, and to test the action and efficacy of anti-cancer drugs at various time points during disease progression.

In this Review, we describe different GEMMs of pancreatic cancer and their utility for understanding the progression of PDAC and for identifying therapeutic targets. We focus in particular on one of the most widely used GEMMs of pancreatic cancer, the LSL-*Kras^G12D^;* LSL-*Trp53^R172H^;* *Pdx1-cre* (KPC) model, and its use in the preclinical testing of anti-cancer agents. Finally, we discuss the importance of GEMMs in translating research findings to the clinic, and highlight their limitations and potential opportunities for their improvement.

## GEMMs of pancreatic cancer: unravelling cancer mechanisms

Many GEMMs of pancreatic cancer have been created (see Table 1) and several feature the deletion or introduction of a mutation into a relevant tumour suppressor gene, often in the context of an activating mutation in *Kras* (see Box 1). Although mutations in *Kras* are a requisite event in the initiation of pancreatic disease, additional genetic events are required to induce tumour formation. Knockout studies have been conducted in the context of mutant *Kras*, with and without additional tumour suppressor mutations, revealing the role of these additional mutations in pancreatic tumour development. These studies have shown, for example, that mutations in *Trp53* and loss of the *Cdkn2a* locus or *Smad4* accelerate PDAC development in the context of mutant *Kras* (see Box 1), but with differing histologies (Aguirre et al., 2003; Bardeesy et al., 2006; Izeradjene et al., 2007). Monoallelic *Trp53* loss accelerates tumour development with the same kinetics as mutant *Trp53^R172H^*, but mutant *Trp53^R172H^* also drives metastatic behaviour in pancreatic tumours (Morton et al., 2010c). When *Brca2* (see Box 1) is deleted in the presence of an activating *Kras* mutation, PDAC formation is suppressed owing to chromosomal instability and apoptosis (Rowley et al., 2011). However, when *Trp53* and *Brca2* are both deleted, mice are more likely to develop PDAC, at reduced latency (Rowley et al., 2011). Moreover, in the absence of functional p53, *Brca2* deletion can cooperate with activated *Kras* mutation to drive pancreatic tumorigenesis (Morton et al., 2011; Rowley et al., 2011; Skoulidis et al., 2010). These studies in GEMMs highlight that human PDAC-associated genetic mutations drive PDAC progression, in cooperation with mutant *Kras*, and are not simply bystander mutations.

**Table 1.**
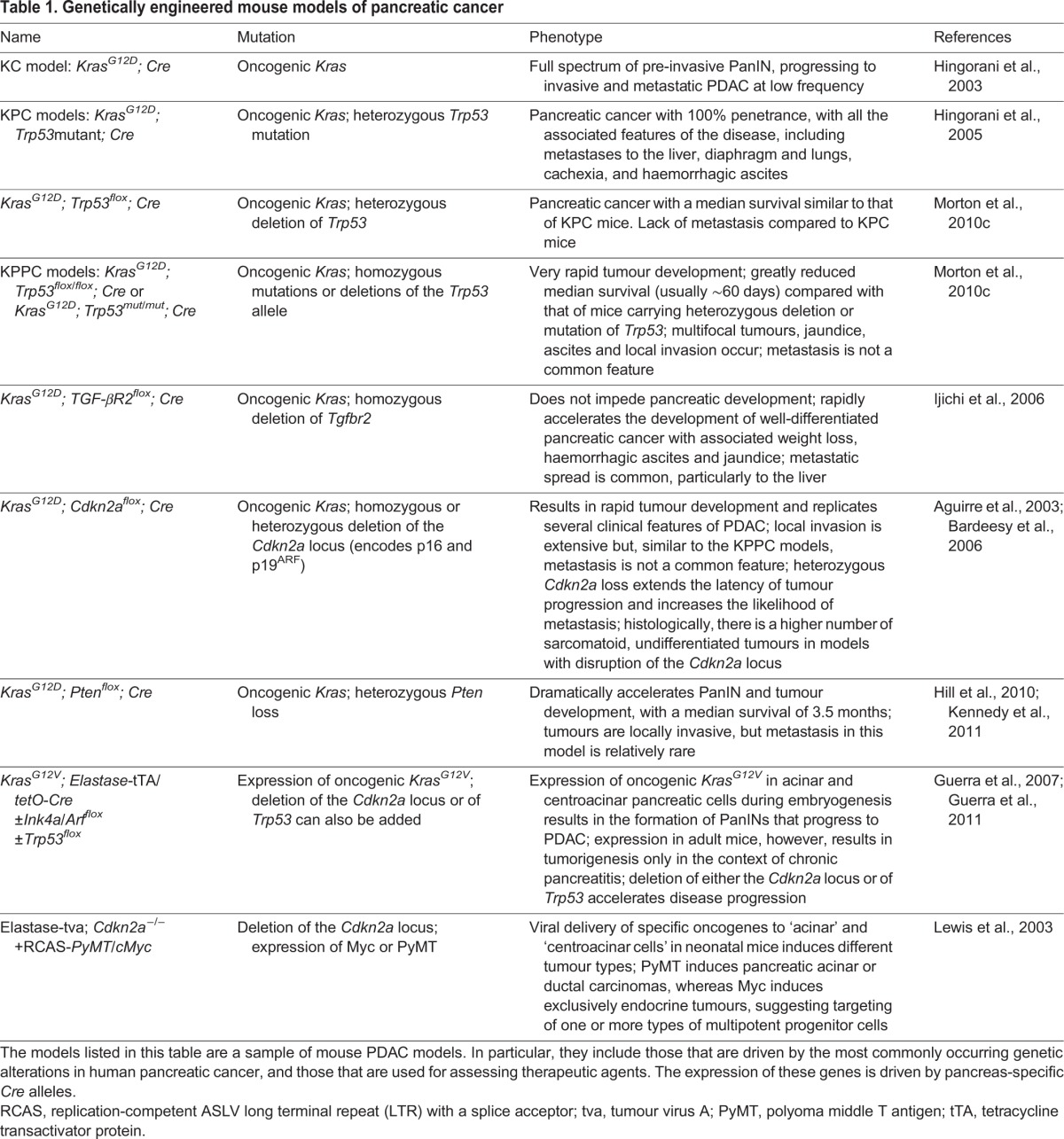
**Genetically engineered mouse models of pancreatic cancer**

In addition to the genes mentioned above, several proteins and pathways have been identified that either promote or suppress PDAC progression, and their effects on tumour development have been studied, some of which are summarized in Table 2. Other GEMMs have shed light on the development of different histological subtypes of the disease. For instance, cystic lesions of the pancreas, such as intraductal papillary mucinous neoplasms (IPMNs) and mucinous cystic neoplasms (MCNs), occur in humans and can progress to invasive cancer if untreated. GEMMs have shown that loss of transcriptional intermediary factor 1 gamma (TIF1γ) or brahma-related gene-1 (Brg1) results in cystic lesions in the pancreas (Vincent et al., 2009; von Figura et al., 2014). TIF1γ is a nuclear protein, the molecular function of which is poorly understood, with existing evidence suggesting that it might regulate TGFβ signalling positively and negatively (Dupont et al., 2005; Dupont et al., 2009; He et al., 2006). Brg1 is the catalytic ATPase component of the SWI/SNF chromatin remodelling complexes, and is therefore involved in gene transcriptional regulation, with evidence suggesting that it acts as a tumour suppressor in a variety of human cancers (Wong et al., 2000; Izeradjene et al., 2007; Vincent et al., 2009; von Figura et al., 2014). Although candidate gene approaches such as those described above have provided valuable information about the associations between individual genes and specific PDAC phenotypes, forward genetic screens such as those based on the use of transposon-mediated mutagenesis (see Box 2) have also helped to identify previously unknown pathways of potential relevance in tumour development in an unbiased manner (Mann et al., 2012; Perez-Mancera et al., 2012).

**Table 2.**
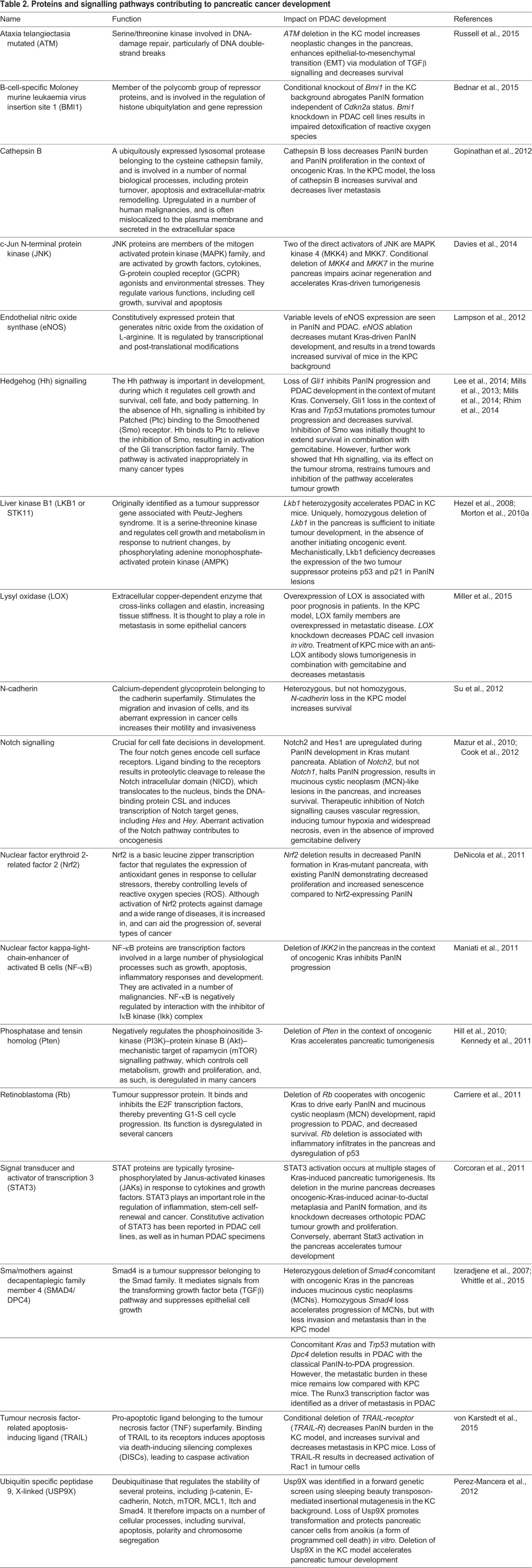
**Proteins and signalling pathways contributing to pancreatic cancer development**

Despite their advantages, these models are not without some limitations. For example, mutant genes may be expressed in the entire pancreas through the use of embryonic pancreatic promoters such as *Pdx-1* and *p48* (Hingorani et al., 2003, 2005; Westphalen and Olive, 2012), but the leakiness of these promoters may result in off-target pathologies in other tissues (Gades et al., 2008; Hingorani et al., 2003, 2005). There is, therefore, a need to develop improved GEMMs of PDAC that overcome such limitations. Recently, an inducible *Kras^G12D^* model has been developed that allows for the conditional and reversible expression of oncogenic *Kras* in the pancreas (Collins et al., 2012a; Ying et al., 2012). This model has been used to study the role of activated Kras in maintaining established tumours, and the mechanisms by which it acts (Collins et al., 2012a,b; Ying et al., 2012). Indeed, results obtained from this model have identified the activation of Yap1 (Yes-associated protein 1) – a transcriptional co-activator in the Hippo pathway that controls cell proliferation, apoptosis and thus organ size, and is frequently overexpressed and activated in different cancers (Zhang et al., 2014) – as a potential bypass mechanism to overcome the dependence of PDAC on oncogenic Kras (Kapoor et al., 2014). Although this model is useful, it is important to note that the inducible Kras is encoded by a transgene, therefore resulting in an extra copy of *Kras* not driven from the endogenous promoter.

Another recently published model has made use of the alternate Flp-*FRT* recombinase system (see Box 2). Thus far, the genetic studies carried out in GEMMs of PDAC have involved germline knockouts or Cre-dependent alleles that are expressed/deleted at the same time as the initiating oncogenic events. These approaches interfere with tumour initiation and progression; therefore, preventative rather than therapeutic approaches are modelled by gene modulation. The Flp-*FRT* recombinase system will enable the generation of GEMMs in which the activation or deletion of genes of interest is under the control of different enzymes, and therefore more amenable to individual manipulation (DeCant et al., 2014; Schönhuber et al., 2014). Indeed, Kras activation and *Trp53* deletion can be temporally separated in the pancreas using a combination of the Cre-*lox* and Flp-*FRT* systems. In *Pdx1-Flp;* *FSF-Kras^G12D/+^;* *FSF-R26^CAG−CreERT2/+^;* *Trp53^lox/lox^* mice (KPF), the deletion of *Trp53* 2 months after the expression of oncogenic Kras in the pancreas induces rapid multifocal tumour development (Schönhuber et al., 2014). The Flp-*FRT* system was also used to show that Pdpk1 (3-phosphoinositide-dependent protein kinase 1) deletion in mutant-Kras-expressing pancreata blocks PanIN progression (Schönhuber et al., 2014). An alternative method for generating mouse models uses the CRISPR/Cas (clustered regularly interspaced short palindromic repeats/CRISPR-associated proteins) gene-editing system (see Box 2) to mutate tumour suppressor genes directly *in vivo*, thereby obviating the need for embryonic stem cell targeting (Sanchez-Rivera et al., 2014; Xue et al., 2014). This approach has recently been demonstrated in the pancreas, where CRISPR-mediated targeting of liver kinase B1 (*Lkb1*) in mice led to tumour growth in conjunction with oncogenic Kras, phenocopying the effect of genetic deletion of *Lkb1* (Chiou et al., 2015). In this study, the authors also induced PDAC development in the murine pancreas by administering adenoviral-Cre and lentiviral-Cre to express oncogenic Kras and delete *Trp53* (Chiou et al., 2015), rather than by the widely used transgenic or knock-in Cre alleles mentioned above*.*

GEMMS have also proven their utility in preclinical settings. In particular, they can be used to study how particular genetic lesions influence responses to therapies, thereby potentially identifying specific patient populations that might benefit from treatment. For instance, our group has used the *Kras; Pten* mouse model, in which tumours have high levels of mTOR (mammalian target of rapamycin) signalling, to test the efficacy of an mTOR inhibitor (Morran et al., 2014). Inhibitors of mTOR signalling have failed in clinical trials of all-comers in pancreatic cancer, where patients are not selected based on the presence of specific mutations. However, cases of efficacy have been reported when patients have mutations in the mTOR pathway (Morran et al., 2014), and our findings support these results and suggest that specific patients might benefit from treatment with these inhibitors. Inhibition of hedgehog signalling, a pathway involved in the generation of the tumour stroma, has been studied in the *Kras; Ink4/Arf^flox^; Cre* model, in which it increases survival (see Table 1) (Feldmann et al., 2008). In addition, the *Kras; Tgfbr2^flox^; Cre* pancreatic cancer model (see Table 1) has been used to assess the efficacy of the EGFR (epidermal growth factor receptor) inhibitor erlotinib, as well as the effect of cancer-associated fibroblast depletion. The depletion of cancer-associated fibroblasts accelerates pancreatic cancer development and decreases survival in this model (Miyabayashi et al., 2013; Ozdemir et al., 2014). The response to a given therapeutic or genetic intervention might vary in PDAC models carrying different genetic alterations. For instance, EGFR ablation prevents tumour development in the background of *Cdkn2a* deletion, but only delays it when p53 is lost (Navas et al., 2012), and erlotinib in *Kras; Tgfbr2^flox^; Cre* mice increases survival in combination with gemcitabine, as described above (Miyabayashi et al., 2013). Taken together with the studies mentioned above, the use of GEMMs carrying different genetic alterations to assess therapeutic targets and agents might be a useful approach to identify subsets of patients who are likely to respond to specific therapies.

The above is a very brief summary of some of the studies done using GEMMs of pancreatic cancer (Guerra and Barbacid, 2013), because an exhaustive discussion of this subject is beyond the scope of this Review. In the next sections, we describe in greater detail the KPC model and its use in preclinical settings because it represents the most common GEMM of PDAC used in this context. We discuss important insights that have emerged from such studies, as well as their clinical relevance and limitations.

## KPC model: its uses for testing novel cancer therapies

Traditionally, KPC mice are generated by the concomitant expression of oncogenic *Kras^G12D^* and of *Trp53* harbouring a conditional point mutation (*Trp53^R172H^*), both driven by a pancreas-specific *Cre*, the *Pdx1-cre* transgene, which is expressed in all cells of the pancreas from an early stage of embryonic development (Fig. 1A). KPC mice were first described in 2005 (Hingorani et al., 2005). These mutant mice develop the complete spectrum of pre-invasive PanIN, as well as end-stage pancreatic cancer (Fig. 1B-G) with 100% penetrance and with a much shorter latency relative to models that express oncogenic *Kras^G12D^* alone (Hingorani et al., 2003). The KPC model also exhibits the clinical features of advanced disease, including loss of body conditioning resembling cachexia, haemorrhagic ascites (see Box 2), and metastases to the liver, lungs, peritoneum and lymph nodes (Hingorani et al., 2005). Histopathologically, the tumours tend to be highly stromal with dense desmoplasia and a high degree of chromosomal instability, but sarcomatoid and anaplastic histologies also occur (see Box 2) (Hingorani et al., 2005). A single mouse can have a tumour with different histological components but this considerable intra- and inter-tumour heterogeneity recapitulates that seen in the human disease. As with other GEMMs, the KPC model is a useful tool to advance our understanding of pancreatic cancer biology, particularly given its genetic and histological similarity to the human disease. In addition, it is probably the most widely used of all GEMMs in evaluating preclinical therapeutic agents. In this Review, the term ‘KPC’ is used to refer primarily to mice harbouring the *Trp53^R172H^* mutation and the *Pdx1-cre* transgene as described above. However, different *Trp53* mutations, such as *Trp53^R270H^*, and other pancreas-specific *Cre* alleles, such as *Ptf1a-Cre* (also called *p48-Cre*), can also be used to drive tumour development in the pancreas.

**Fig. 3.**
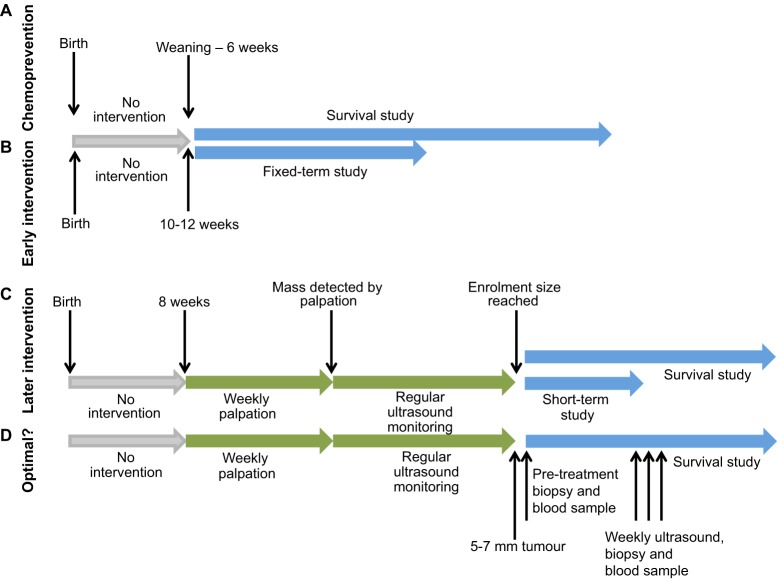
**Enrolment scheme for chemoprevention and intervention studies in KPC mice.** In preclinical studies using LSL-*Kras^G12D^;* LSL-*Trp53^R172H^; Pdx1-Cre* (KPC) mice, different approaches are used to address different clinical questions. Grey arrows indicate no intervention; green arrows indicate pre-treatment monitoring; and blue arrows indicate treatment assessment. (A) Chemoprevention studies aim to evaluate dietary compounds or therapeutic agents that prevent tumour initiation or slow/arrest tumour development. Mice are enrolled between weaning and 6 weeks of age and, at this stage, usually present with early-stage PanIN. Treatments can be assessed at pre-determined time points or can continue until end point (to evaluate survival). (B) In early intervention studies, which are used to test anti-metastatic therapies, treatment is initiated when mice are 10-12 weeks old, when they commonly have early and late PanINs and occasional tumours. Treatment can last for a fixed period or can continue until end point. (C) Later intervention studies are performed on animals bearing established tumours and are thus relevant for identifying treatments that can reverse, slow or arrest cancer once fully established. These studies require more elaborate monitoring of mice, including manual palpation and ultrasound to monitor tumour size and progression. Treatment can begin when tumours reach the enrolment size for a study. Depending on tumour size, treatment can be short (9-11 days) or long (up to 45 days) (see main text for more detail). (D) Optimal design for intervention studies in KPC mice, incorporating serial sampling to allow pre- and post-treatment assessments of tumour and blood samples.

In this section, we discuss how the KPC model is utilised in both chemopreventive and interventional settings, which are designed to address different clinical questions (Fig. 3). Chemoprevention studies aim to evaluate the effects of dietary compounds or therapeutic agents that can prevent tumour initiation or that can slow or arrest tumour development. They also include epidemiological studies to identify factors that can increase or reduce the risk of developing cancer. By contrast, interventional studies are designed to evaluate the effect of a treatment – or treatment combinations – on tumour progression and metastasis (early intervention studies) or on established tumours (late intervention studies). They are thus relevant for identifying treatments that can reverse, slow or arrest cancer once it is fully established.

### Chemoprevention

Several chemoprevention studies have been conducted in the LSL-*Kras^G12D^; Pdx1-Cre* (KC) model, in which oncogenic Kras alone is expressed in the pancreas, as well as in KPC mice. In this setting, mice with early-onset pancreatic disease (generally consisting of early-stage PanIN) are treated very early and prior to the onset of final-stage PDAC. In published studies, the age at enrolment varies from weaning to 10 weeks in KC mice and from weaning to 6 weeks in the KPC model, and the effect of a treatment is assessed either at pre-determined time points (to evaluate the effect on cancer initiation) or at the disease end point (to evaluate the effect on overall survival) (Bai et al., 2011; Chugh et al., 2012; Husain et al., 2013; Keenan et al., 2014; Lampson et al., 2012; Li et al., 2013; Liao et al., 2013; Morton et al., 2010b; Plentz et al., 2009; Yip-Schneider et al., 2013). For example, KPC mice have a lower tumour burden and increased survival time when treated from 4 weeks of age with atorvastatin, a cholesterol-lowering competitive inhibitor of 3-hydroxy-3-methylglutaryl coenzyme-A (HMG CoA) reductase (Liao et al., 2013). A similar outcome was seen when treatment was initiated at 5 weeks of age with sulindac, a non-steroidal anti-inflammatory drug (NSAID), which is an inhibitor of aldo-keto reductase family member 1B10 (AKR1B10), a protein that is overexpressed in human PDAC (Li et al., 2013). The favourable results reported in these two studies might have been caused by the inhibition of PanIN progression. However, later disruption of tumour development and maintenance, or a continuous effect throughout the disease process, cannot be ruled out and further work is needed to dissect these effects. Epidemiological experiments have also been performed in the KPC model and in other GEMMs, including analysis of the effect of diet, nicotine and aspirin on tumour development (Hermann et al., 2014; Lanza-Jacoby et al., 2013; Philip et al., 2013). Nicotine administration accelerates Kras-induced PanIN progression in the pancreas, with increased proliferation in PanIN in nicotine-treated mice. Mechanistically, nicotine results in the activation of Akt-ERK-Myc signalling and the subsequent downregulation of Gata6 (Hermann et al., 2014). Intermittent and chronic calorie restriction in the KC model decreases PanIN progression, with fewer PanIN-2 and PanIN-3 lesions observed. Proliferation in PanIN was also significantly reduced as a consequence of calorie restriction (Lanza-Jacoby et al., 2013). Conversely, a high-fat diet increased stromal content and accelerated pancreatic tumour progression, with cyclooxygenase-2 (Cox2) playing an important role in this process (Philip et al., 2013).

### Early intervention

Early intervention studies are carried out on mice of a fixed age, without predetermining whether or not they have advanced tumours. Mice are generally enrolled on a study between 10-12 weeks of age (Plassmeier et al., 2013). At this stage, most KPC mice in a cohort would not have advanced PDAC but instead a mix of early and late PanINs and occasional tumours. Treatment in this setting generally occurs for longer periods of time relative to treatment in late intervention studies, and can continue until the disease end point, or for a fixed period of time after which the mice are allowed to age with no further intervention. These early intervention studies thus provide a means to test for drugs that can prevent metastases formation. This is not possible using late intervention protocols, when metastases have already developed. Xenograft ‘metastasis’ models also cannot be used for such tests because they do not recapitulate the stroma that characterises pancreatic cancer, which likely contributes to the metastatic phenotype.

Early intervention studies can also provide insight into the pathways that are important for PDAC initiation and development, and, additionally, can help us to understand whether the early perturbation of signalling pathways affects metastasis. For example, the inhibition of Src kinase with Dasatinib, a drug used in some forms of leukaemia, with treatment beginning in 70-day-old KPC mice until disease end point, significantly decreased the formation of metastases, albeit without an effect on primary tumour growth or overall survival. Thus, although it might not prevent primary tumour progression, Src inhibition represents a potential anti-metastatic strategy for treating PDAC (Morton et al., 2010b). In another study, treatment of 70-day-old KPC mice with a LOX-blocking antibody decreased tumorigenesis in combination with gemcitabine, and decreased metastatic burden (Miller et al., 2015). Treatment of KPC mice from 8 weeks of age with the smoothened inhibitor IPI926 demonstrated that long-term inhibition of hedgehog signalling actually accelerates tumour development and decreases survival (Rhim et al., 2014), whereas treatment of KPC-*Brca1* mice at the same time point with the DNA demethylating drug 5-aza-2′-deoxycytidine (decitabine) significantly inhibited tumour growth (Shakya et al., 2013). However, one important consideration is that early intervention studies involve extended treatment, often prior to the existence of frank carcinoma. Tumours developing under these circumstances might evolve to circumvent inhibition, and therefore might be different molecular and biological entities to the tumours that form in untreated mice. Therefore, a favourable outcome in this setting does not indicate that the therapy will successfully inhibit an established tumour, and experiments with promising agents might need to be repeated in a late intervention setting.

Given that many pancreatic cancer patients die from distant metastases even after surgical removal of primary tumours (Heinemann and Boeck, 2008), it is important to test anti-metastatic therapies under these conditions. Thus, we and others are now trialling the excision of primary tumours from the pancreas of mice in order to improve our testing of anti-metastatic therapies; these studies are still at an early stage.

### Later intervention

As previously mentioned, at the time of diagnosis, individuals with PDAC usually present with late-stage carcinoma. Thus, in evaluating a novel cancer therapy or therapy combination, it is important to assess efficacy on already established tumours, either in terms of survival or by clinical and molecular parameters. In preclinical settings, this requires the identification of tumour-bearing mice prior to the initiation of treatment. The KPC model has a variable latency, which necessitates the use of manual palpation and non-invasive imaging modalities, both to identify animals that carry tumours and to determine tumour size. The schema in Fig. 3C outlines the typical monitoring and screening of KPC mice in late intervention studies (Sastra and Olive, 2013). Beginning at approximately 2 months of age, mice are manually palpated weekly to detect any masses in the abdomen and, with experience, tumours as small as 2 mm or less can be identified by this method. When a mass is detected by palpation, high-resolution ultrasound is used to confirm the presence of a tumour and to measure its size. Ultrasound can also be used to follow tumours over the course of treatment, and volumetric measurements can be performed to establish whether tumour growth is altered in response to therapy (Sastra and Olive, 2013). Treatment can begin when tumours reach a size that makes a mouse eligible for enrolment into a late intervention study.

Several therapeutic studies have been published using KPC mice with different approaches to target tumours (Beatty et al., 2011; Cook et al., 2012; Courtin et al., 2013; Frese et al., 2012; Jacobetz et al., 2013; Neesse et al., 2013; Olive et al., 2009; Provenzano et al., 2012). Mice enrolled in these studies had varying tumour sizes, between 2-10 mm in diameter, with intervention beginning when tumours are 2-5 mm, 4-6 mm, 6-9 mm or 5-10 mm for individual studies published. The Hedgehog (Hh) and Notch signalling pathways are two developmental pathways that are activated in pancreatic cancer. When these signalling pathways are inhibited in the KPC model, in combination with treatment with gemcitabine, the mice have improved survival relative to controls, although the mechanism of action differs between the two pathways. The inhibition of Hh signalling decreased the stromal content of KPC tumours, effectively increasing the delivery and/or efficacy of gemcitabine (Olive et al., 2009). By comparison, the inhibition of Notch signalling seemed to induce vascular regression, causing tumour hypoxia and widespread necrosis, even in the absence of improved gemcitabine delivery (Cook et al., 2012).

Targeting the stromal component, either by depleting the extracellular matrix component glycosaminoglycan hyaluronic acid (HA) or by inhibiting the matrix protein CTGF (connective tissue growth factor), was also effective in combination with gemcitabine. Whereas HA depletion by PEGPH20 resulted in improved vasculature and increased gemcitabine delivery, blocking CTGF decreased the expression of the pro-survival protein XIAP (X-linked inhibitor of apoptosis) and induced the killing of tumour cells (Jacobetz et al., 2013; Neesse et al., 2013). The depletion of HA described for the treatment of larger tumours has also been tested in tumours of 2-5 mm diameter, and has similarly improved survival in combination with gemcitabine (Provenzano et al., 2012). PEGPH20 is currently being assessed in a randomized Phase 2 clinical trial assessing its efficacy as a first-line therapy against metastatic pancreatic cancer in combination with gemcitabine/nab-paclitaxel compared to gemcitabine/nab-paclitaxel alone (https://clinicaltrials.gov/ct2/show/NCT01839487). PEGPH20 initially proved problematic clinically, with a high rate of blood clots and other thromboembolic events observed in the PEGPH20 arm. Following a protocol amendment, the interim data from the trial was recently revealed to be promising, with increased median progression-free survival and overall response rate in the PEGPH20 arm compared with the gemcitabine/nab-paclitaxel arm. There was also a trend towards improvement in median overall survival (http://www.halozyme.com/Investors/News-Releases/; News Release on 31st May 2015).

In another preclinical study, the efficacy of nab-paclitaxel was assessed in combination with gemcitabine in a limited-duration experiment (Frese et al., 2012). In this study, nab-paclitaxel effectively altered gemcitabine metabolism by decreasing the levels of the primary gemcitabine-metabolizing enzyme, cytidine deaminase (CDA). This increased gemcitabine stability and, uniquely, it induced tumour regression (Frese et al., 2012).

Another approach to targeting PDAC involves the immune system. Administration of AMD3100, a C-X-C chemokine receptor type 4 (CXCR4) inhibitor, in combination with the inhibitory checkpoint antagonist anti-PD-L1 (anti-programmed death 1 ligand 1), results in the loss of p53-positive tumour cells and in the accumulation of CD3^+^ T-cells (see Box 2) in the tumour area (Feig et al., 2013). In another study, the immune system was modulated using a CD40 agonist. The resulting tumour shrinkage was mediated by macrophages, and the expected influx of T cells into the tumour did not occur (Beatty et al., 2011). Further work showed that tumour-derived granulocyte-macrophage colony stimulating factor (GM-CSF) regulates the recruitment of Gr-1^+^CD11b^+^ myeloid cells, which suppress antigen-specific T-cell responses (Bayne et al., 2012). These studies exemplify a few of the many approaches that are being considered in the targeting of PDAC, including combinatorial approaches that target tumour cells and the individual components of the stroma.

Established tumours in the KPC model undergo rapid growth. With tumours of 5-10 mm diameter, the median survival of untreated mice is around 9-11 days. As a consequence, treatment regimens tend to be of a limited duration, even where combination treatments induce a statistically significant increase in survival. Short-term studies can also be carried out with fixed durations of treatment. These enable in-depth mechanistic analyses of the therapeutic effects of a given treatment, but might also be useful in cases where longer-term treatment is not feasible. Nab-paclitaxel, for instance, is formulated using human albumin and induces anaphylaxis in mice, thereby necessitating short-term treatment (Frese et al., 2012). In general, using smaller tumours tends to lengthen the treatment period and consequently drug exposure. For instance, with tumours of 2-3 mm diameter, the median survival of untreated mice is approximately 45 days (Provenzano et al., 2012). This approach permits the long-term effects of drug exposure to be assessed, both on the tumour and on the host, which is not possible in mice with larger tumours. This also models the condition of individuals who present at an early stage at the clinic.

### Factors that influence response to therapy

Irrespective of tumour size, interventional approaches are generally labour- and resource-intensive, requiring a large mouse colony and a substantial investment of time to screen and monitor treated animals. Particularly in the case of large tumours, mice are also lost to ill health prior to enrolment, thereby extending the enrolment period, and in fixed-duration experiments, mice that do not reach the required time point owing to short survival times need to be replaced.

**Fig. 4.**
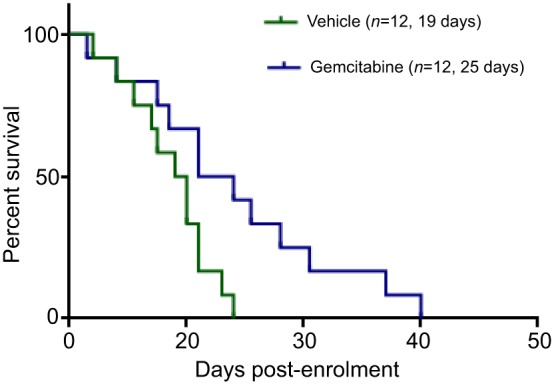
**Effects of tumour size on response to treatment in the KPC model.** Gemcitabine imparts a small yet significant survival benefit in mice with smaller tumours (our unpublished observations). Mice were enrolled on study when tumours reached 3-6 mm mean diameter. Mice were treated with either saline or gemcitabine (100 mg/kg body weight) intraperitoneally twice per week until end point. 12 mice were enrolled per cohort. The median survival post-enrolment is indicated for each cohort in the figure. The Log-rank test was conducted using GraphPad Prism (*P*=0.0236).

When assessing responses to therapeutic agents, varying results can be obtained depending on tumour size. There is a lack of studies examining the differences in tumour response based on initial tumour size. However, our preliminary observations suggest that, in KPC mice with tumours of 6-9 mm diameter at the time of enrolment in the study, the tumour growth in the first 7 days post-enrolment correlates with survival, perhaps indicating that 7 days is a useful time point to assess early responses to treatment. As another example, there is generally not a significant difference in survival between vehicle- and gemcitabine-treated cohorts in KPC mice with large tumours (Olive et al., 2009). However, when mice with smaller tumours (3-6 mm mean diameter) are treated with gemcitabine, and compared to vehicle-treated controls, gemcitabine seems to have a beneficial effect on their survival (Fig. 4). This might be due to poor drug perfusion in large tumours because of their well-developed desmoplastic stroma (Olive et al., 2009). Another example of differing outcomes is seen with the use of the matrix-depleting agent PEGPH20, which was independently assessed in the two studies involving mice with large or small tumours mentioned above (Jacobetz et al., 2013; Provenzano et al., 2012). In combination with gemcitabine, PEGPH20 improved survival in both circumstances but with some differences. Smaller tumours (2-5 mm diameter) were characterized by a significant remodelling of their stroma, including a depletion of fibrillar collagen and α-smooth muscle actin (α-SMA)-positive fibroblasts. By contrast, the stromal content of larger tumours at end point remained similar between the control and treated groups. These findings suggest that primary tumour burden is not the sole determinant of treatment outcome and that this outcome can be influenced by the presence of a well-established tumour stroma. Other factors that might affect therapeutic outcome and survival include tumour location, extent of metastatic disease and occurrence of cachexia (Bachmann et al., 2008; Neoptolemos et al., 2004; Watanabe et al., 2004), which together reflect the complex and multifaceted nature of advanced pancreatic cancer.

Duration of treatment can also affect therapeutic outcome. As previously described, Hh pathway inhibition was first reported to decrease stromal content in KPC mice with large tumours, thereby increasing gemcitabine delivery to tumours and extending survival when administered in combination with gemcitabine (Olive et al., 2009). Unfortunately, despite a promising Phase 1 clinical trial, these results were not borne out by the Phase 2 study. Following the failure of this clinical trial, further work was carried out in the KPC model to understand the discrepancy. In this study (Rhim et al., 2014), mice were treated for an extended period of time, and the preclinical data recapitulated what was seen in the clinic, with inhibition of Hh signalling decreasing survival rather than improving it. This indicates that the prolonged inhibition of signalling pathways might have different effects to those intended, which would not be picked up by a ‘large tumour’ intervention study (Rhim et al., 2014). Given the shorter treatment duration in mice with large tumours, apparent treatment outcomes might simply be indicative of acute responses of tumours to therapy (in the case of Hh inhibition, the initial depletion of the stroma and the corresponding increase in gemcitabine delivery to tumours). In reality, extended exposure to compounds might be required to unveil the consequences of a treatment's indirect effects or the development of resistance through the modulation of signalling or by other mechanisms.

The genetic background and the specific genetic alterations of the mice that are used in a study are two other factors to consider when evaluating response to therapy. As has been discussed earlier, the outcome of genetic and therapeutic studies can vary depending on the underlying genetic alterations in the mouse models. Understanding these differences, and identifying cohorts that are likely to respond to a given therapy, might inform the selection of patient populations in clinical trials.

### Translating mechanistic information from mice to humans: limitations and opportunities

Improving success rates in clinical trials depends on the use of robust and predictive preclinical models. Owing to its genetic and histopathological similarity to human PDAC, the KPC model is relevant for evaluating therapies and for understanding treatment mechanisms. However, the examples mentioned above illustrate the importance of determining the best way of using preclinical models, so that the obtained results accurately reflect clinical outcome. Results obtained from studies using the KPC model suggest that mice bearing smaller tumours might be of particular relevance for survival studies because their use allows sufficient time for adverse effects to become apparent. Care must also be taken when interpreting the results of such studies, in particular focusing on change in tumour volume and not absolute tumour size.

Another important question that requires consideration is whether the tumour at the end of the treatment period is the same biological entity as the initial tumour at the start of the study. Until now, tumour comparisons have been static and carried out between treatment cohorts (e.g. vehicle versus drug) because it has not been possible to obtain pre- and post-treatment tumour samples. Recently, however, a laparotomy (see Box 2) method has been developed that allows tumour biopsies to be obtained surgically (Sastra and Olive, 2014) from KPC tumours. This technique allows the paired comparison of pre- and post-treatment samples, for example, to analyze whether the continued accumulation of mutations alters the activity of signalling pathways targeted by drugs. This technique might also enable biopsies to be obtained and examined prior to, during and after treatment, and then compared to determine how a tumour is modulated by treatment and whether it remains the same entity in terms of its histopathology and signalling pathways. Although this approach might remove the need to use large cohorts to account for inter-tumour heterogeneity and biological variation, small individual biopsies might not be representative of the entire tumour due to heterogeneity.

A key strength of preclinical models is the ability to gain mechanistic insight into the tested therapies, in a manner that would not be possible in a clinical setting. For example, fixed-time-point pharmacodynamic studies can be conducted, allowing the immediate (24-48 h), intermediate (7 days) and long-term effects of treatment to be compared, for example on signalling pathways and tumour characteristics such as proliferation, apoptosis, etc. Therapies that target metastasis can be tested in early and advanced disease, and the effect of drugs on organs other than the pancreas can be assessed. Haematological and biochemical analyses can complement molecular investigations both in pharmacodynamic and survival studies. Routine imaging including high-resolution ultrasound as discussed above, but also magnetic resonance imaging (MRI), positron emission tomography and micro-computed tomography, can be carried out to evaluate tumour progression and dissemination. Preclinical testing in GEMMs also has the potential to identify tumour biomarkers that can be used to either predict drug response or to stratify patients for treatment (Singh et al., 2012).

In addition to targeting the primary tumour and disseminated disease, studies can be conducted on symptoms, such as cachexia, which are associated with PDAC. The importance of the stroma and immune compartments in tumours can also be investigated. Recent work has, in fact, shown that the stromal compartment in PDAC might have a role in suppressing pancreatic tumours (Ozdemir et al., 2014; Rhim et al., 2014); however, careful interpretation of data is required when tumours are initiated in the absence of stroma, or where depleting the stroma results in a substantial inflammatory response. As mentioned above, there are also several approaches for targeting the immune system that are being explored to enhance the anti-tumour immune response, such as activation of CD40, inhibition of chemokine (C-X-C motif) ligand 12 (CXCL12) and vaccines (Beatty et al., 2011; Feig et al., 2013; Keenan et al., 2014). Given that chemotherapy is a mainstay of PDAC treatment, combinations with chemotherapy should be considered when assessing new drugs preclinically. Indeed, most preclinical work to date has focused on the use of gemcitabine in combination with various agents. The changing landscape of treatment in the clinic necessitates the need for a more up-to-date approach to chemotherapy in the preclinical models. The FOLFIRINOX regimen might be challenging to model in mice, but new therapies can be tested in combination with gemcitabine/nab-paclitaxel to further develop current treatments.

Despite their advantages, GEMMs have several drawbacks, including the length of time needed to generate mutant mice carrying several genetic alterations. In conditional GEMMs, such as the KPC model, genetic alterations are often activated simultaneously in a large number of cells during development in the mouse, even though they are used as models of sporadic, non-inherited human cancers. In addition, models such as KPC mice cannot be used to study the cell-of-origin of pancreatic cancer. This has required the use of alternative promoters, such as the inducible tetracycline-inducible Elastase-*cre* or the Nestin promoter (Guerra and Barbacid, 2013). Tissue-specific promoters, such as *Pdx1-cre*, are sometimes expressed in other tissues, resulting in off-target pathologies, such as papillomas and lymphoma. Tumour development in GEMMs can also take a long time and occurs with variable latency. Unlike xenografts, tumour monitoring might require advanced imaging, including high-resolution ultrasound and MRI. Although useful information can be obtained from these imaging modalities, they require specialist training and equipment that is not always easily available. In all, preclinical testing in GEMMs is significantly more expensive than the testing performed in *in vitro* culture or xenografts. Nevertheless, their advantages, as discussed here, make them a very promising preclinical platform, and a potential means of assessing complex treatment modalities and of identifying anti-cancer drug combinations to evaluate in the clinic.

## Conclusions

Owing to their similarity to human disease, KPC mice and other GEMMs of pancreatic cancer can be excellent tools to assess therapeutics and to understand mechanisms of drug action and resistance in tumours. They have not yet been completely validated in terms of their ability to predict the outcome of trials; however, as discussed in this Review, there is extensive evidence of their relevance in preclinical research (Singh et al., 2010). In order to remain clinically relevant, preclinical testing in these models must keep abreast of developments in the clinical sphere. GEMMs might also provide information relevant for patient stratification in clinical trials. If we are to derive benefit from these models, consideration must be given to the way in which they are used, including the timing and scheduling of treatment, because this might affect experimental outcomes and therefore the predictive accuracy of the model. Any insight gained from such preclinical models must be extended to the clinic to demonstrate their relevance. The models that are currently available are relevant; however, they will become more predictive of clinical outcomes as we gain more knowledge and understanding of the various factors that affect response to therapy. In the future, it is likely that more attention will be paid to the model used in preclinical testing, the stage of intervention and, importantly, what constitutes a response that is robust enough to provide confidence for translation to the clinic.

## Notes

This article is part of a subject collection on Model Systems in Drug Discovery: from Bench to Patient. See related articles in this collection at http://dmm.biologists.org/cgi/collection/drugdiscovery
